# Label-free and Non-destruction Determination of Single- and Double-Strand DNA based on Quantum Weak Measurement

**DOI:** 10.1038/s41598-018-38454-x

**Published:** 2019-02-13

**Authors:** Tian Guan, Yuxuan Yang, Qianwen Zhang, Yonghong He, Naihan Xu, Dongmei Li, Lixuan Shi, Yang Xu, Xiangnan Wang

**Affiliations:** 1 Department of Biomedical Engineering, School of Medicine, Tsinghua University, Beijing, 100084 China; 2 Shenzhen Key Laboratory for Minimal Invasive Medical Technologies, Institute of optical imaging and sensing, Graduate School at Shenzhen, Tsinghua University, Shenzhen, 518055 China; 3 Department of Physics, Tsinghua University, Beijing 100084, China Shenzhen maternity & child healthcare hospital, Shenzhen, 518055 China; 4 Key Lab in Healthy Science and Technology, Division of Life Science, Graduate School at Shenzhen, Tsinghua University, Shenzhen, China

## Abstract

The process of unwinding and renaturation of DNA has been widely used in studies of nucleotide sequence organization. Compared with traditional methods for DNA unwinding and renaturation, the label-free and non-destruction detection technology is significant and desiderated. We realized an optical system based on optical rotation *via* weak measurement for detection of single- and double-strand state of DNA. The optical rotation, which was induced by the status change of single and double DNA strands, was exploited to modulate the preselected polarization of a weak measurement system. With this modulation, the optical rotation caused by the separation of DNA strands can be determined through the center wavelength shift of the output spectrum. By monitoring the wavelength shift in real time, the separation processes of the DNAs with different base ratio (25% and 70%) and length (4nt and 40nt), and DNAs with three terminally modified cholesterol molecules were experimentally explored in varied pH and temperature conditions. In addition, the detection limit of the DNA concentration was obtained to be 5 × 10^−6^ mol/L. Our work based on optical rotation detection of single- and double-strand DNA exhibits the unique advantages of real-time monitoring, label-free, non-destruction and simplicity.

## Introduction

**Figure 1:**
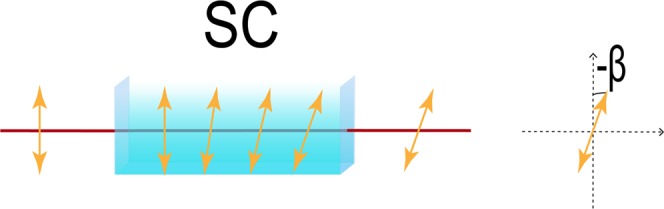
Optical rotation changes. The figure shows that (1) when the optical rotation of the liquid in the sample cell (SC) is small, the pre-selection angle will be changed to a lesser degree, and (2) when the optical rotation of the liquid in the SC is large, the pre-selection angle will be largely changed.

The use of DNA as recognition ligands for hybridization- and aptamer-based bioassays has attracted great attention in bioanalysis such as real-time PCR detection^1^, genomes^2^, and the like. The ssDNA or dsDNA state will change rapidly with the experimental environment. This feature places high demands on the real-time nature and sensitivity of detection. For detection, more sensitive methods have been developed; for example, gold nanoparticles (Au NPs)^3–5^, fluorescence^6,7^, electrochemical methods^8,9^, and atomic mass spectrometry. However, these methods can destroy the original DNA and cannot be detected in real time. It is very inconvenient to observe the experimental process of DNA. Weak measurement is a detection method developed in the past two years that is suitable for high-sensitivity detection. It has great potential for the detection of biological molecules and reactions^10^. Our previous amino acid experiments have demonstrated the feasibility and high measurement accuracy of this method. In the application of optical rotation detection, the resolution obtained by our method is 2.138 × 10^−5^ degree^10^, about 2 orders of magnitude higher than the standard polarimeter. Unlike reflection and refraction structures, this transmission system is not affected by refractive-index changes and remains effective in complex environments, independent of temperature. The change of ssDNA and dsDNA state is often achieved by means of a change in temperature. The optical rotation of DNA is relatively constant up to 75 °C, and then decreases smoothly to essentially zero rotation at about 100 °C, indicating completion of the helix-to-coil transition. It is indicated that DNA will generate optical rotation changes in the state of high-temperature untwisting. Therefore, the changes in the state of DNA are also examined by examining the changes in optical activity^11^. Changes in the optical rotation of DNA can change the pre-selection angle of our measurement system, which is shown in Fig. 1. Changing the ssDNA and dsDNA states of the DNA solution in the sample cell (SC) will change the optical activity, and thus the light passing through the SC will change the pre-selection angle. The solution with large optical rotation will greatly change the pre-selection angle, and a solution with a small optical rotation changes the pre-selection angle to a lesser degree. This weak measurement scheme provides a new method for the study of the ssDNA and dsDNA states. It has the advantages of high precision, robustness, simple operation, and fast response. It can be detected in real time and is untagged and without damage.

In this work, a transmission-based optical rotation detection method based on weak measurement is proposed, which can be used to amplify weak signals, and has been introduced into many high-precision measurements^12–17^. In this system, the pre-selected polarization state is changed using the optical activity of the DNA liquid during the change of the single- and double-strand states. We monitored the movement of the center-wavelength output spectrum to analyze the optical activity of the single- and double-strand states of different DNAs (ssDNA for single-strand DNA and dsDNA for double-strand DNA). As is known, DNA can be untwisted and renatured using different methods. A pro-denatured nucleic acid denaturing agent can be a strong acid, strong base, monohydric alcohols, urea, AgCl, DMSO, betaine, and formamide, or even one or more of the foregoing. When the environment is restored, the ssDNA is renatured to dsDNA. Experiments have demonstrated the feasibility and high measurement accuracy of the system. The assay consisted of six types of DNA: self-extracted pcDNA3.1 (+) plasmid, DNA of the same base ratios of 40nt and 4nt in length, 20% and 70% base DNA of the same 20nt chain length, and DNA with three terminally modified cholesterol molecules. The determination of the material provides a high degree of discrimination, can detect the status of ssDNA and dsDNA stably and effectively, and has differentiating degrees for different bases and different lengths. The detection limit of the solution concentration is as low as 5 × 10^−6^ mol/L, and the detection performance is better than the weak measurement in the time domain. Unlike reflection and refraction structures, the transmission system is not affected by refractive-index changes and remains effective in complex environments. External real-time monitoring and lossless labeling have no effect on DNA solutions. In summary, this weak measurement scheme provides a new method for the study of DNA molecule denaturation, with advantages of high accuracy, robustness, simple operation, and fast response.

In the weak optical measurement experiment, we use the horizontal polarization $|{\rm{H}}\rangle$ and the vertical polarization $|{\rm{V}}\rangle$ to represent the system’s two orthogonal eigenstates^18^. Left- and right-circularly-polarized light are expressed by $|{\rm{L}}\rangle =\frac{\sqrt{2}(|H\rangle -{\rm{i}}|V\rangle )}{2}$ and $|R\rangle =\frac{\sqrt{2}(|H\rangle +{\rm{i}}|V\rangle )}{2},|H\rangle$ and $|V\rangle$ can also be represented by left- and right-circularly-polarized light as $|H\rangle =\frac{\sqrt{2}(|L\rangle +|R\rangle )}{2}$ and $|V\rangle =\frac{\sqrt{2}i(|L\rangle -|R\rangle )}{2},$ respectively. Since our device, the relationship between the measurement system and the measurement device is presented in a weakly coupled form, some minor shifts between the two eigenstates occur. In the time domain, this shift refers to the space coordinates, while it represents the momentum in the frequency domain. When the system adjusts to the appropriate pre-selection $|{{\rm{\psi }}}_{i}\rangle$ and then selects $|{{\rm{\psi }}}_{f}\rangle$, the system can amplify the shift between the two eigenstates, which in turn can read the result from the output pointer. We defined *A*_*ω*_ by ${A}_{\omega }=\frac{\langle {{\rm{\psi }}}_{f}|A|{{\rm{\psi }}}_{i}\rangle }{\langle {{\rm{\psi }}}_{f}|{{\rm{\psi }}}_{i}\rangle }$, and take *A*_*ω*_ as an amplifying factor in the expression of the amplification of weak value^18^.

**Figure 2:**
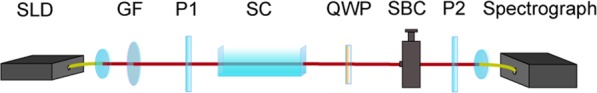
Experimental setup. Superluminescent laser diode (SLD830S-A20,830 nm,Inphenix). QWP (quarter-wave plate) as light source. Double linear polarizers (ThorlabsInc.,LPVIS050-MP,extinction ratio of 100000:1) as P1 and P2 for pre- and post-selections. SC, Sample cell for holding experimental DNA solution. SBC, Soleil-Babinet compensator for phase selection.

In this work,

We designed a weak measurement scenario with a linear optical system in the frequency domain, which accuracy greatly exceeds standard interferometry. The system schematic diagram of weak measurement is shown in Fig. 2. Devices used in this work are the following:

Superluminescent diode (SLD), Thorlabs Inc., SLD830S-A20

Gaussian filter (GF)

Linear polarizer (P1) and (P2),Thorlabs Inc., LPVIS050-MP2, extinction ratio of 100000:1

Sample cell (SC), a 30-mm-long cuvette

Quarter-wave plate (QWP)

Soleil-Babinet compensator (SBC), Thorlabs Inc., SBC-IR

The experimental light generated by SLD is first processed by GF and then preselected by P1. In the system, SC was used as a container. Light pre-selection is processed by QWP with P1. At the appropriate operating point position, SBC can introduce an initial phase difference between horizontal and vertical polarization components, coupled with P2 for post selection. After the beam is filtered by the P2, the output light beam collected by the spectrograph is analyzed by computer.

Incident light with the center wavelength of *λ*_0_ (corresponding to momentum *P*_0_) passed through a GF followed by P1, whose optical direction pointed at an angle of *θ* with the vertical direction^18^. The QWP was exploited in the system with an angle of π/4, producing the pre-selection in collaboration with P1. Then, the initial state of the system can be expressed as

1 $$|{\psi }_{in}\rangle =\frac{\sqrt{2}}{2}{e}^{i\theta }|L\rangle +{e}^{-i\theta }|R\rangle $$

and the pre-selection can be calculated by

2 $$|{\psi }_{pre}\rangle =(|H\rangle \langle H|+|L\rangle \langle L|)|{\psi }_{i}\rangle =\frac{\sqrt{2}}{2}(-{e}^{-i\theta }|H\rangle +i{e}^{-i\theta }|V\rangle )$$

A phase difference ${\delta }_{0}+\delta (pH)$ between $|{\rm{H}}\rangle$ and $|{\rm{V}}\rangle$ was induced by the SBC, which could modify the working point in a measurement, and *δ*_0_ was the initial phase difference. *δ*(*pH*) is related to the pH of the solution. The pointing angle of P2 used for the post-selection operation is −π/4, which can be represented as

3 $$|{\psi }_{post}\rangle =\frac{\sqrt{2}}{2}[-{e}^{i({\delta }_{0}+\delta (pH))/2}|H\rangle +{e}^{-i({\delta }_{0}+\delta (pH))/2}|V\rangle ].\,$$

We set $${\rm{\eta }}=({\delta }_{0}+\delta (pH))/2.$$

The final state of the system can be expressed as:

4 $$|{\psi }_{final}\rangle =(|L\rangle \langle L|+|R\rangle \langle R|)|{\psi }_{post}\rangle =\frac{1}{2}(i{e}^{-i\eta }-{e}^{i\eta })|L-\frac{1}{2}(i{e}^{-i\eta }+{e}^{i\eta })|R\rangle $$

The observable of this system can be presented as

5 $$A=\frac{1}{2}[|V\rangle \langle V|-|H\rangle \langle H|].$$

Then we can get the weak value by the following equation

6 $${A}_{\omega }=\frac{\langle {{\rm{\psi }}}_{f}|A|{{\rm{\psi }}}_{i}\rangle }{\langle {{\rm{\psi }}}_{f}|{{\rm{\psi }}}_{i}\rangle }=i\frac{1}{2}\,\tan [\frac{\pi }{4}-\frac{({\delta }_{0}+\delta (pH))/2}{2}-\theta ].$$

The shift of momentum can be described as ${\rm{\delta }}P=2{\rm{k}}{({\rm{\Delta }}P)}^{2}Im{A}_{\omega }$^19^. According to our previous work^13^, we can find the wavelength shift as follows:

7 $$\delta \lambda =-\,\frac{4\pi {({\rm{\Delta }}\lambda )}^{2}}{{\lambda }_{0}}Im{A}_{\omega }=-\,\frac{2\pi {({\rm{\Delta }}\lambda )}^{2}\,\tan [\frac{\pi }{4}-\frac{({\delta }_{0}+\delta (pH))/2}{2}-\theta ]}{{\lambda }_{0}}.$$

As shown in Equation (7), the phase difference between the center wavelength shift of the output spectrum and the preselected angle is given, and the quantitative relationship between them is given, which plays an important role in finding the optimum operating point for the most sensitive detection. This relationship can be clearly displayed by the theoretical fitting, as shown in Eq. (7). *β*_0_ is the initial phase difference, which is critical for the measurement of working point.

The SBC is used to introduce the phase difference between H polarization and V polarization, adjusted to the most sensitive and most suitable measured phase. By maintaining a constant phase difference, pre-selected angles can be detected. Since the pre-selected polarizer angle has a quantitative relationship with the center-wavelength shift of the output spectrum, as shown in Eq. (6), a sample cell can be added between the first polarizer and the QWP, and the ssDNA and dsDNA solutions in different states will change the pre-selected angle. The proper angle is available

8 $$[{\rm{\alpha }}]=100\alpha /{\rm{l}}\cdot {\rm{C}}.$$

As shown in Equation (8), [α] represents the corresponding rotation of the solution, α is the optical activity corresponding to the rotation angle, the interaction length is 3 cm inour system, and C represents the solution concentration. Therefore, different states and concentrations of solutions in the SC may produce different optical rotations, so that small changes in the solution can be detected by the center wavelength shifting of the output spectrum. It is well known that dsDNA will unwind into ssDNA in an extremely acidic or extremely alkaline environment or at temperatures above 95 °C for more than 4 min. When the environment is at room temperature and neutral pH, complementary ssDNA sequences will automatically bind to dsDNA. In order to conduct cross-validation experiments, we first untwisted DNA by changing the acidity and alkalinity of the DNA solution, then used heated and cooled methods to unwind and renaturate the DNA, and then place custom standard single-strand DNA complementary sequences into the SC. And stir it to make it renatured. The platform was used to test the central wavelength of the DNA solution in different environments, all in line with dsDNA unspooling to reduce the center wavelength, ssDNA renaturation to increase the center wavelength.

We demonstrate the effect of the weak measurement system by optical rotation response experiment. First, it was verified that the system can qualitatively detect changes in the acid-base environment to de-rotate the DNA molecule. The pcDNA3.1(+) plasmid solution was put into the neutral environment at a concentration of 0.2723 g/L in the sample cell SC and a strong alkali solution added. In the NaOH solution, the pH of the DNA solution was adjusted to 12.89, and the DNA was completely untwisted by stirring until the signal was stable. Sulfuric acid solution was then added, and the pH was adjusted back to neutral at 5.18. After stirring, the DNA was completely renatured. Then, the acid was added to adjust the pH to 1.03. The pH was adjusted to 12.31 by stirring the NaOH solution, and the DNA was completely unwind by stirring. Adding sulfuric acid solution to adjust the pH to 0.92, and stirring were carried out to completely unwind the DNA.

**Figure 3:**
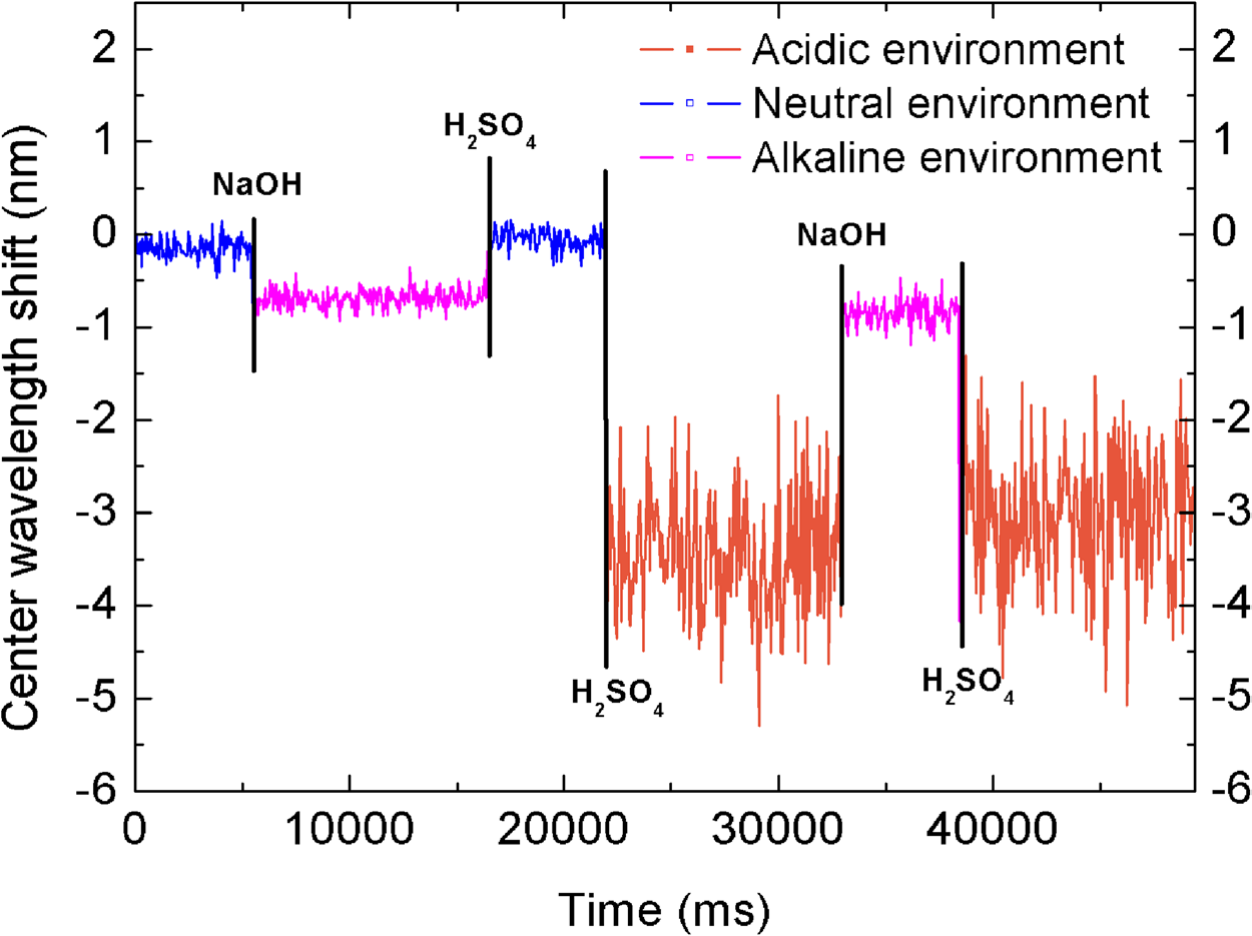
Systematic response of ssDNA and dsDNA states at different pH values.

Each time an acidic or alkaline solution is added to change the pH, the signal is stable (the result is shown in Fig. 3) until the concentration drops to 5 × 10^−6^ mol/L. The amount of shift in the center wavelength of the DNA under different types of pH conditions will be significantly different. The literature shows the same phenomenon as the acidic DNA status shown in Fig. 3; at ionic strengths below 0.001, the intrinsic viscosity of single-strand DNA is greater than that of double-strand DNA^20^, which means that the viscosity of DNA will increase during unspooling and the light transmittance will decrease. Hence, the rate of single-strand status-signal passing is reduced and it appears to be larger in our system. Under the acidic environment, the unwinding will be fuller, and the center-wavelength shift of the DNA in an acidic environment is larger than in an alkaline environment.

Cross-validation was then performed, and it was verified that the system can qualitatively detect temperature changes to unscrew the DNA molecule. Silicone oil was heated in a container with a heating plate, and a 0.28 -g/L solution of pcDNA3.1(+) was placed in the centrifuge tube and into the silicone oil. After the silicone oil was heated to 110 °C for 10 min, the centrifuge tube was removed and DNA added to the SC. The signal was collected and the center-wavelength offset was calculated.

**Figure 4:**
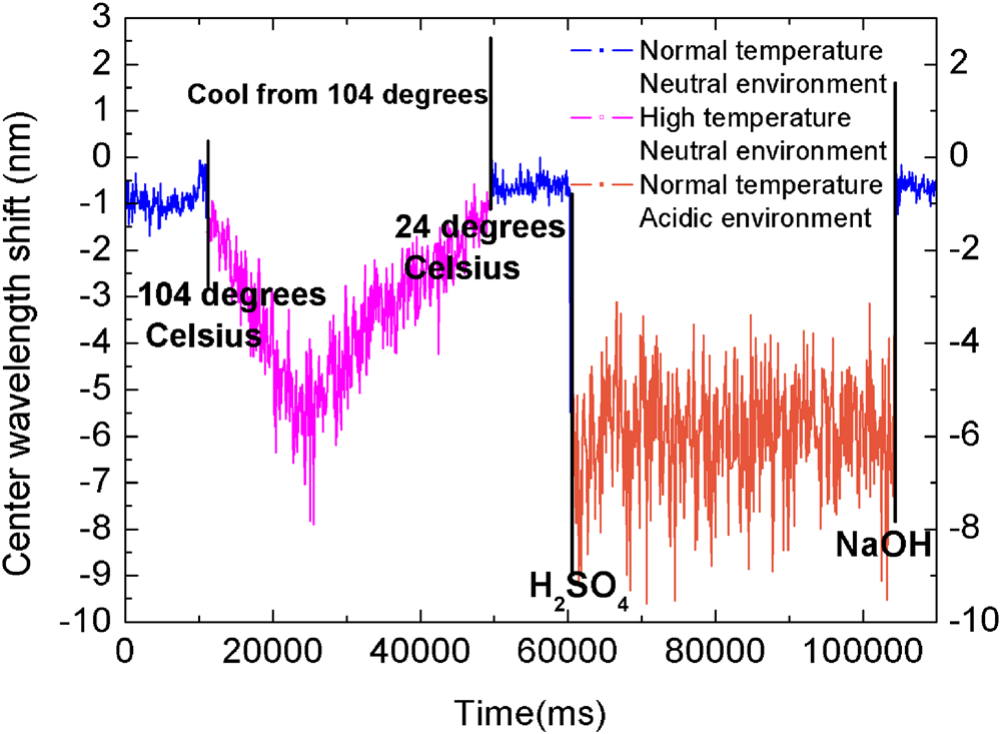
Systematic response of ssDNA and dsDNA states during DNA cooling and contrast of center-wavelength shift between temperature unwinding and extreme pH unwinding.

As shown in Fig. 4, the DNA is in the SC, and the center-wavelength first decreases and then increases; that is, the DNA is always unwinding at temperatures above the renaturation temperature and begins to refold after the temperature first drops to the renaturation temperature.

**Figure 5:**
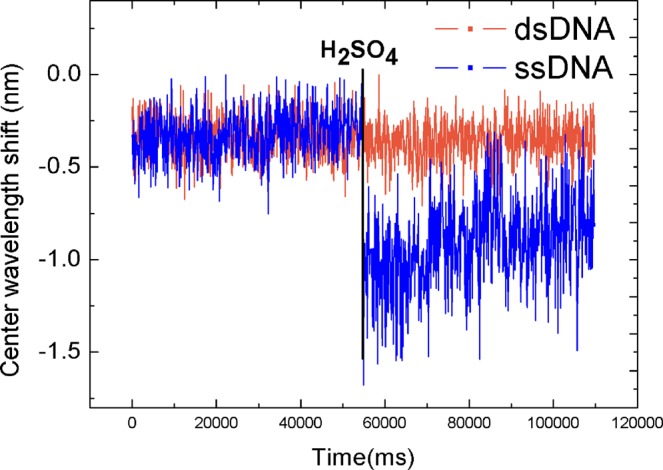
Comparison of changes in ssDNA and dsDNA states at extreme pH values.

To verify that the DNA is affected by the change of the optical rotation produced by the unwinding of the double strand, we put the customized standard ssDNA into the SC, added the sulfuric acid solution, and recorded the results after stabilization. We then added the standard dsDNA into the SC, added the same concentration and volume of sulfuric acid solution, and recorded the results after stabilization. The results are shown in Fig. 5. When the pH of the dsDNA is reduced to acidic, the center wavelength decreases as the DNA unwinds. When the pH of the double-strand DNA is reduced to acidic, there is no significant change in the center wavelength because there is no unwinding process.

**Figure 6:**
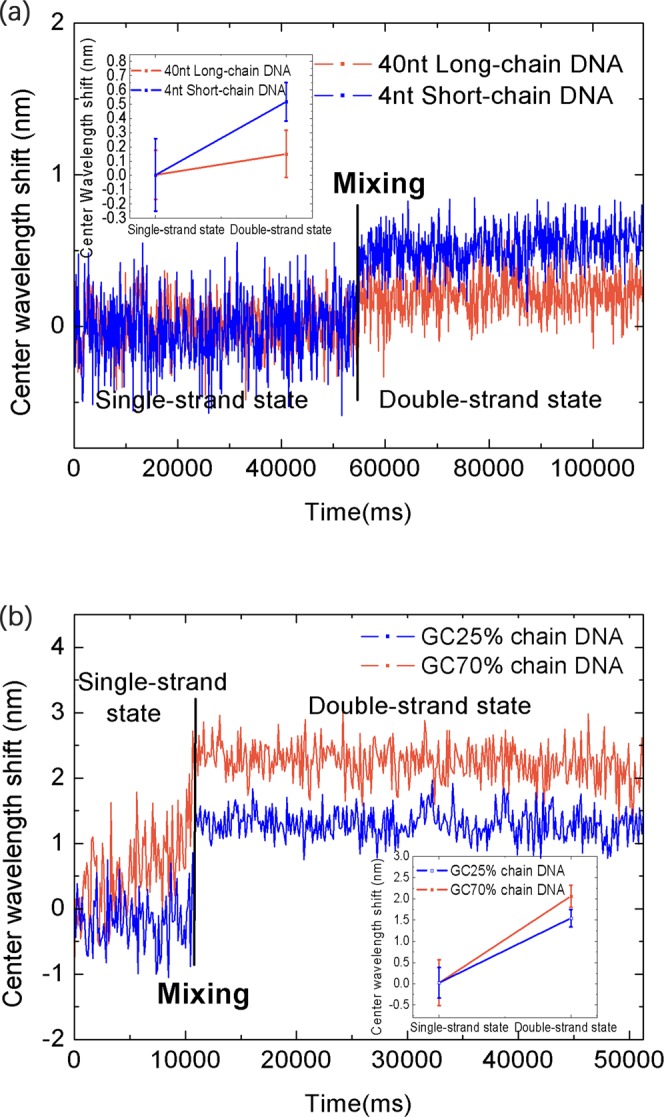
Comparison of different DNA changes in ssDNA and dsDNA states. (**a**) Center-wavelength shifts of 40nt- and 4nt-long DNA from the ssDNA renaturation to the dsDNA process; (**b**) center-wavelength shift of DNAs with base ratios of 70% and 25% from ssDNA renaturation to the dsDNA process.

We then wanted to determine whether the shift of the center wavelength of the standard DNA unwinding process was related to the length and base ratio of the DNA strand. We added a short 4nt-long DNA single strand and its complementary sequence to the SC, initiated stirring to anneal the two strands, and recorded the change of the center wavelength during the change from single- to double-strand renaturation. Similarly, the same treatment was performed on 40nt-long DNA (base ratio the same as that of 4nt-long DNA). As a result, and shown in Fig. 6(a), the shift of the center wavelength between the untwisted state and the refolded state of the short-strand DNA is greater, which means that, compared to long-strand DNA, the short-strand DNA changes its optical state more dramatically. Subsequently, 20nt-long DNA with a base ratio of 70% and 20nt-long DNA with a base ratio of 20% were processed in the same manner and the center wavelength recorded. As shown in Fig. 6(b), the amount of shift in the center wavelength between the untwisted state and the enatured state of the DNA having a base ratio of 70% is much greater, which means that, compared to the DNA having a base ratio of 25%, the DNA having a base ratio of 75% changes its optical state more dramatically.

**Figure 7:**
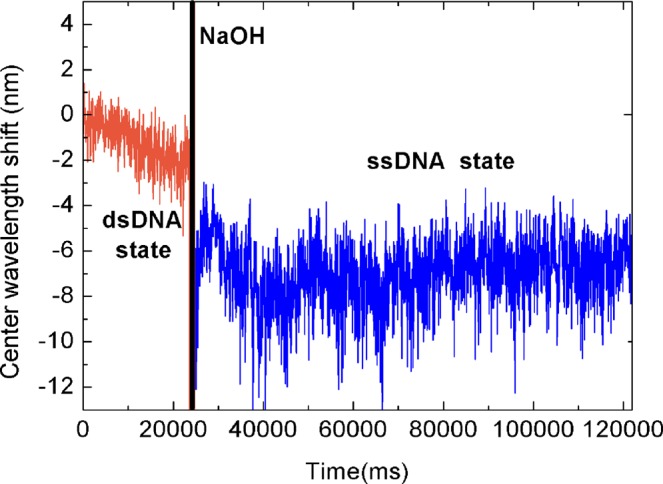
Center-wavelength shift of ssDNA renaturation to dsDNA in DNA with three terminally modified cholesterol molecules.

In practical experimental applications of DNA, there are often studies of the association of DNA with different small molecules. For example, combining Au nanoparticles with DNA sequences to analyze ethanolamines^21^. DNA insertion of small electrochemical molecules can be used as novel electrochemical biosensor^22^. We placed dsDNA modified with small molecules of cholesterol into the SC, and then added NaOH solution into the SC until the neutral environment became alkaline. Then, the dsDNA state will unwind to the ssDNA state in an alkaline environment. The signals of dsDNA and ssDNA were compared in the two different states and the results are shown in Fig. 7. When the dsDNA state turns into the ssDNA state, the center wavelength shifts downwards, in agreement with previous experimental results. Thus, this method can also be used to detect the ssDNA and dsDNA states of DNA chains modified with small molecules.

## Discussion

In this experiment, we conducted some studies on the real-time non-destructive detection of ssDNA and dsDNA states using weak measurements. In different environments, DNA can produce single- and double-strand changes in state. When the DNA changes from the dsDNA state to the ssDNA state, the center wavelength shifts toward the decreasing direction. On the contrary, when the DNA changes from the ssDNA state to the dsDNA state, the center wavelength will shift toward the rising direction. Whether it is in a very acid or alkali environment, or heated to high temperature, the center wavelength will move negatively from dsDNA unwinding into the ssDNA state. In addition, putting the four different custom, pure, single-strand DNA solutions into the sample pool to undergo renaturation, the center wavelength will move in the positive direction. These experimental results strongly support the feasibility of using the proposed DNA measurement method in weak measurement systems. The method is also applicable to the detection of the state of DNA strands modified with small cholesterol molecules.

We conducted 14 experiments and the actual lowest concentration detected was 5 × 10^−6^ mol/L. When the concentration of the solution drops to 5 × 10^−6^ mol/L, the accuracy of the DNA solution cannot be guaranteed. It is worth noting that with increasing interaction length the solution can be improved, depending on the expression of Eq. (7).

The proposed method has the advantages of non-destructive labellessness, high precision, simple operation, strong stability, strong robustness, fast response, and low cost, among other advantages. High-precision detection of ssDNA and dsDNA states with different lengths and different base ratios plays an important role in the bioassay of hybrid aptamers. The method described in this work can be used to detect various extracted plasmids, standard chains, and modified small-molecule DNA strands. Real-time detection of ssDNA and dsDNA status is important for the use of DNA as a recognition ligand for hybridization- and aptamer-based bioassays.

## Methods

### Materials

95% H_2_SO_4_ and NaOH were purchased from TianJin BaiShi Chemical Co., Ltd (China). All the standard DNA sequences were purchased from ShanghaiSangon Biotech (China). A plasmid kit for DNA extraction was purchased from Shenzhen Pufei Shengnuo Technology Co., Ltd. The result of the extraction detected no protein or RNA contamination.

DNA sequences used in this work are the following:

ssDNA:

ATGC (4nt long)

GGCCTTTCTACTCGTGGAAGGGTTCGAAGATCAGCTATCC (40nt long)

CGTCCGTCGAGCAGCTGCGA (70% GC base ratio)

ATGTTACATATCTAGCTAAT (25% GC base ratio)

Complementary sequence of ssDNA (auto-refolding to double strand with complementary sequence in neutral temperature environment):

GCAT (4nt long)

GGATAGCTGATCTTCGAACCCTTCCACGAGTAGAAAGGCC (40nt long)

TCGCAGCTGCTCGACGGACG (70% GC base ratio)

ATTAGCTAGATATGTAACAT (25% GC base ratio)
